# Diffusion Tensor Imaging of Epileptogenic Lesions in TSC

**DOI:** 10.15844/pedneurbriefs-30-2-3

**Published:** 2016-02

**Authors:** Divakar Mithal, Maura Ryan

**Affiliations:** 1 Division of Neurology, Ann & Robert H. Lurie Children's Hospital of Chicago, Chicago, IL; and Departments of Pediatrics and Neurology, Northwestern University Feinberg School of Medicine, Chicago, IL; 2 Division of Medical Imaging, Ann & Robert H. Lurie Children's Hospital of Chicago, Chicago, IL; and Department of Pediatrics, Northwestern University Feinberg School of Medicine, Chicago, IL

**Keywords:** Surgical Epilepsy, Tuberous Sclerosis, Diffusion Tensor Imaging

## Abstract

Investigators from University of California Los Angeles, studied whether epileptogenic tubers in Tuberous Sclerosis Complex (TSC) can be identified by diffusion tensor imaging (DTI).

Investigators from University of California Los Angeles, studied whether epileptogenic tubers in Tuberous Sclerosis Complex (TSC) can be identified by diffusion tensor imaging (DTI). The group used retrospective analysis of 23 pediatric patients with TS who underwent epilepsy surgery. This population had extensive pre-operative data including video EEG, MRI, and PET scans. MRI and PET data were co-registered. Magnetic dipoles were also identified in some patients. Surgical decisions were made by a multidisciplinary group that included epileptologists, neurosurgeons, neuroradiologists and psychologists. Tubers were considered epileptogenic if there was a “meaningful reduction” in seizure frequency after resection.

By using specific diffusion tensor parameters such as axial diffusion (AD), apparent diffusion coefficient (ADC), fractional anisotropy (FA) and restricted diffusivity (RD) the authors show a difference in diffusivity values between epileptogenic and non-epileptogenic regions, specifically for ADC and RD. The methods used include an expanded area beyond the tuber itself, termed the “peri-tubular” region, which also shows a significant difference in ADC and RD from non-epileptic pre-tubular regions. Finally, receiver-operator curves were used to establish tuber + peritubular cutoff values for both ADC and RD. Together these imaging findings across 543 tubers, of which 6% were epileptogenic, indicate a potential method for delineating epileptogenic sites in tuberous sclerosis. The study is limited in that it is a retrospective analysis of patients who underwent surgery, and does not discuss the potential epileptogenicity of tubers in non-surgical candidates. [1]

COMMENTARY. TSC is a multi-lesional epileptic disorder, in which not all tubers are epileptogenic. Identifying epileptogenic surgical candidate lesions is critical for any chance of seizure freedom. Current imaging methods that are used in conjunction with electrophysiology include MRI, fMRI, PET, SPECT and MEG. Of these methods, MRI is the most widely available.

The current pre-surgical identification of epileptogenic loci includes a gold standard of intracranial EEG, although this is not routine for TSC due to limited capabilities at surgical centers [3]. FDG-PET and magnetic source imaging may be utilized for pre-surgical evaluation, but both are highly specialized tests with limited availability, even for epilepsy surgical centers [4, 5].

DTI is a powerful MRI tool that uses the movement of water molecules to describe three dimensional aspects of white matter tracts and intracranial structures in more detail than standard MR images [2]. DTI holds promise as a method for better evaluation of three dimensional structures like the tubers of TSC. In particular, a long-standing controversy in TSC is whether the tuber alone is epileptogenic or whether the peritubular region also has epileptogenicity [3].

A multi-disciplinary approach to TSC surgery remains the optimal and safest route to surgical planning, given that no single method adequately proves epileptogenicity of tubers. Although still investigatory, the findings presented by Yogi A. et al [1] are promising in that a widely available methodology may at some point be applicable for pre-surgical evaluation.
